# Supplementary material for: Immunogenicity, Efficacy, and Effectiveness of Two-Dose and Shorter Schedules of Hepatitis E Vaccine: A Systematic Review
Source: Vaccines (Basel). 2024 Dec 31;13(1):28. doi: 10.3390/vaccines13010028 (PMC11768984; doi:10.3390/vaccines13010028)
Supplement: Supplementary file 1 [file vaccines-13-00028-s001.zip › vaccines-3351103-supplementary.pdf]

Supplementary Table S1. List of studies excluded from the final review.

**Author, year; location**	**Brief description**	**Type of data included**	**Reason for exclusion**
**During initial vaccine development**			
Zhang *et al*, 2005; China	Phase I study	Safety	No data on immunogenicity / efficacy
Wu et al, 2012; China	Subset analysis of women who inadvertently received either HEV 239 (n=37) or hepatitis B vaccine (n=31) in the phase III trial (Zhu *et al*, 2010; China)	Safety	No data on immunogenicity / efficacy of HEV 239
Wu *et al,* 2013; China	Subset analysis of 14,065 participants with known HBsAg status at baseline in the Phase III trial (Zhu *et al*, 2010; China)	Safety and immunogenicity	No data on immunogenicity after one or two doses of HEV 239. Participants with fewer than three doses were excluded from analysis.
**Post-licensure studies**			
Yu *et al*, 2019; China	Open labelled controlled trial in elderly participants (>65 y) using 3 dose usual schedule (0,1 and 6 mo)	Safety and immunogenicity	No data on immunogenicity were available after one or two doses of HEV 239. Participants with less than three doses were excluded from analysis.
Zhao *et al*, 2022; China	Double blind trial, with participants randomised to receive 3 doses of either bivalent HPV 16 and 18 or HEV 239 (placebo) at 0,1 and 6 months	Safety and efficacy	Efficacy to prevent high-grade genital lesions by HPV 16 and 18 was noted. No data on efficacy to prevent hepatitis E (either serological or clinical infection)
Unpublished: Clinicaltrials.gov ID NCT02584543; China	Coadministration of HEV 239 with hepatitis B vaccine	Safety and immunogenicity	No data on immunogenicity after one or two doses of HEV 239. Individuals with fewer than three doses were excluded from analysis.
Unpublished: Clinicaltrials.gov ID NCT03365921; China	HEV 239 lot-to-lot consistency	Safety and immunogenicity	No data on immunogenicity after one or two doses of HEV 239. Individuals with fewer than three doses were excluded from analysis.
